# Role of cardiac MRI-based multi-modality imaging in diagnosis and management of patients with cardiac mass

**DOI:** 10.1186/1532-429X-17-S1-P344

**Published:** 2015-02-03

**Authors:** Jiayu Sun, Yucheng Chen, Da Zhu, Senlin Ying, Luo Yong, Tianjing Zhang, Shi Chen, Qing Zhang

**Affiliations:** 1 West China Hosptial, Chengdu, China; 2 MR Collaborations NE Asia, Siemens Healthcare, Beijing, China

## Background

Cardiac mass is a relative rare disease which is associated with remarkable morbidity and mortality while challenging the management strategy. The purpose of this study was to evaluate the cardiac MRI (CMR)-based multi-modality imaging in diagnosis and management of patients with cardiac mass.

## Methods

CMR based multi-modality imaging including echocardiogram, cardiac MRI, as well as PET-CT were performed as for patients with suspected cardiac mass. Management strategy was decided according to patient basic status (age and surgical risk) as well as characteristics of the mass derived from multi-modality imaging including location, morphology, border, metastasis or not, tissue characteristics, blood supply, and the risk of thrombosis.

## Results

From Oct 2011 to Dec 2013, total 62 patients (33 females) diagnosed with cardiac mass through routine trans-thoracic echocardiography were enrolled in this study with mean age 47.7±22.0 years. According to CMR imaging, 39 patients were diagnosed as intra-cardiac neoplasm (27 "benign"+ 11 "malignant"), while 20 were pseudo-tumors (17 "thrombi" and 3 "cysts"). 3 cases within them were reclassified as normal variants. 37 patients were eligible candidates for surgical removal, while 4 patients received further PET-CT scan for characterization of the mass with subsequent extra-cardiac biopsy. Further pathological exam reveal a high accuracy of cardiac MRI in differentiate the benign and malignant tumor (97.5%), as well as neoplasm with pseudo-tumors (100%). As for 21 patients do not received the surgery, 3 died during follow-up, 8 patients with MRI suspected cardiac thrombosis resolved after anti-coagulation therapy. All 10 patients which were considered benign according to the CMR appearance and had low risk of embolization survived with good life quality (mean follow-up time 1.5 years).

## Conclusions

CMR based multi-modality imaging provides a confident risk-stratification and tissue characterization for cardiac mass, which provides good evidence for accurate clinical decision making in patients with suspected tumors.

## Funding

None.

